# Supplementary material for: Structural analysis of the SARS-CoV-2 methyltransferase complex involved in RNA cap creation bound to sinefungin
Source: Nat Commun. 2020 Jul 24;11:3717. doi: 10.1038/s41467-020-17495-9 (PMC7381658; doi:10.1038/s41467-020-17495-9)
Supplement: Supplementary file 1 — Supplementary Information [file 41467_2020_17495_MOESM1_ESM.pdf]

Structural analysis of the SARS-CoV-2 methyltransferase complex involved in RNA cap creation bound to sinefungin

Petra Krafcikova[1], Jan Silhan[1], Radim Nencka[1]*, Evzen Boura[1,]*

[1]Institute of Organic Chemistry and Biochemistry AS CR, v.v.i., Flemingovo nam. 2., 166 10 Prague 6, Czech Republic

*correspondence to nencka@uochb.cas.cz. or boura@uochb.cas.cz

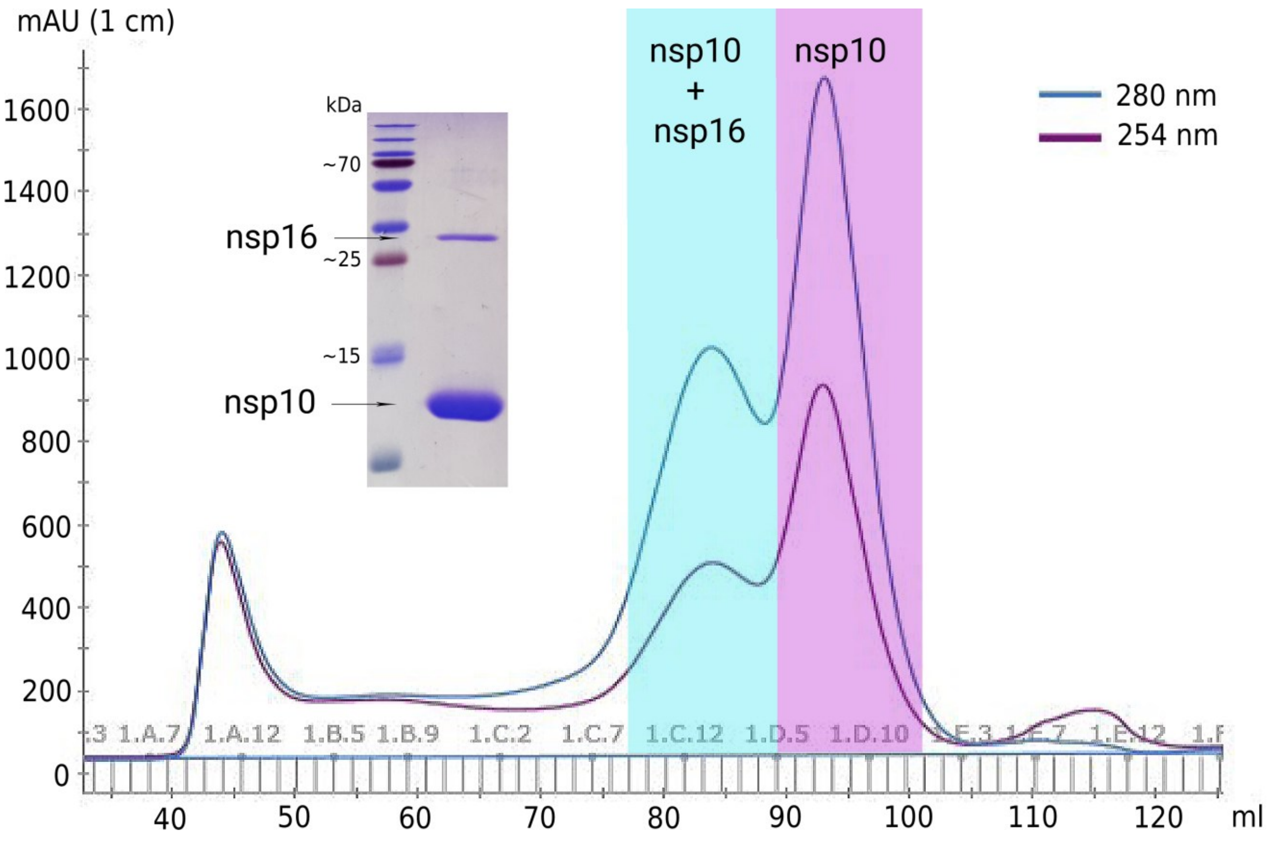

SI Figure 1: Gel filtration profile of the nsp10-nsp16 protein complex

Upon affinity chromatography the nsp10-nsp16 complex was subjected to size exclusion (gel filtration) chromatography (gel filtration was performed only once). The peak corresponding to the nsp10-nsp16 complex (highlighted in cyan) was kept for further experiments. The purified nsp10-nsp16 complex is also shown on an SDS-PAGE gel.

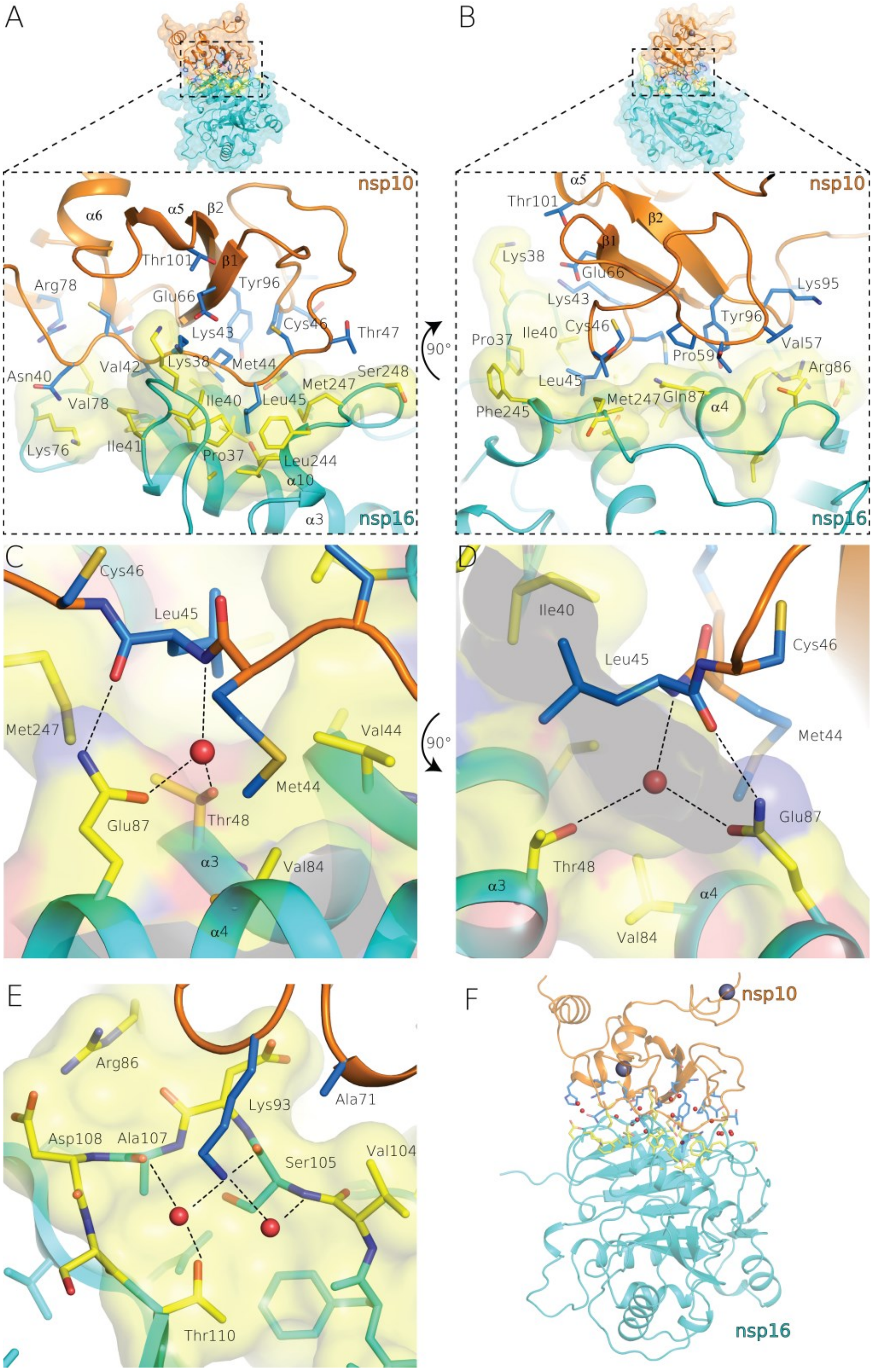

SI Figure 2: Selected interactions at the vast nsp10-nsp16 interface

A-B) Representation of the nsp10-nsp16 complex where interface residues are shown as labeled in blue (nsp10) and yellow (nsp16). The residues involved in the interface interactions are shown as sticks. A) Coiled-coil region of nsp10 (residues 40-47) involved mainly in hydrophobic interactions. B) 90° turn side view for greater perspective of nsp10-nsp16 interface. C-D) Hydrogen bonding of Leu45 (nsp10) main chain connected via hydrogen bonds I) directly to Glu87 and II) to both residues Glu87 and Thr48 mediated though water molecule. E) Side view of the nsp10-16 interface, Lys93 (nsp10) with hydrogen bonds connecting main chain groups from Ser105, Ala107 and side chain form Thr110 of nsp16. F) nsp10:nsp16 entire structure with water molecules involved in the interactions connecting the dimer interface. Waters are shown as red spheres. Hydrogen bonds are shown as dotted lines.

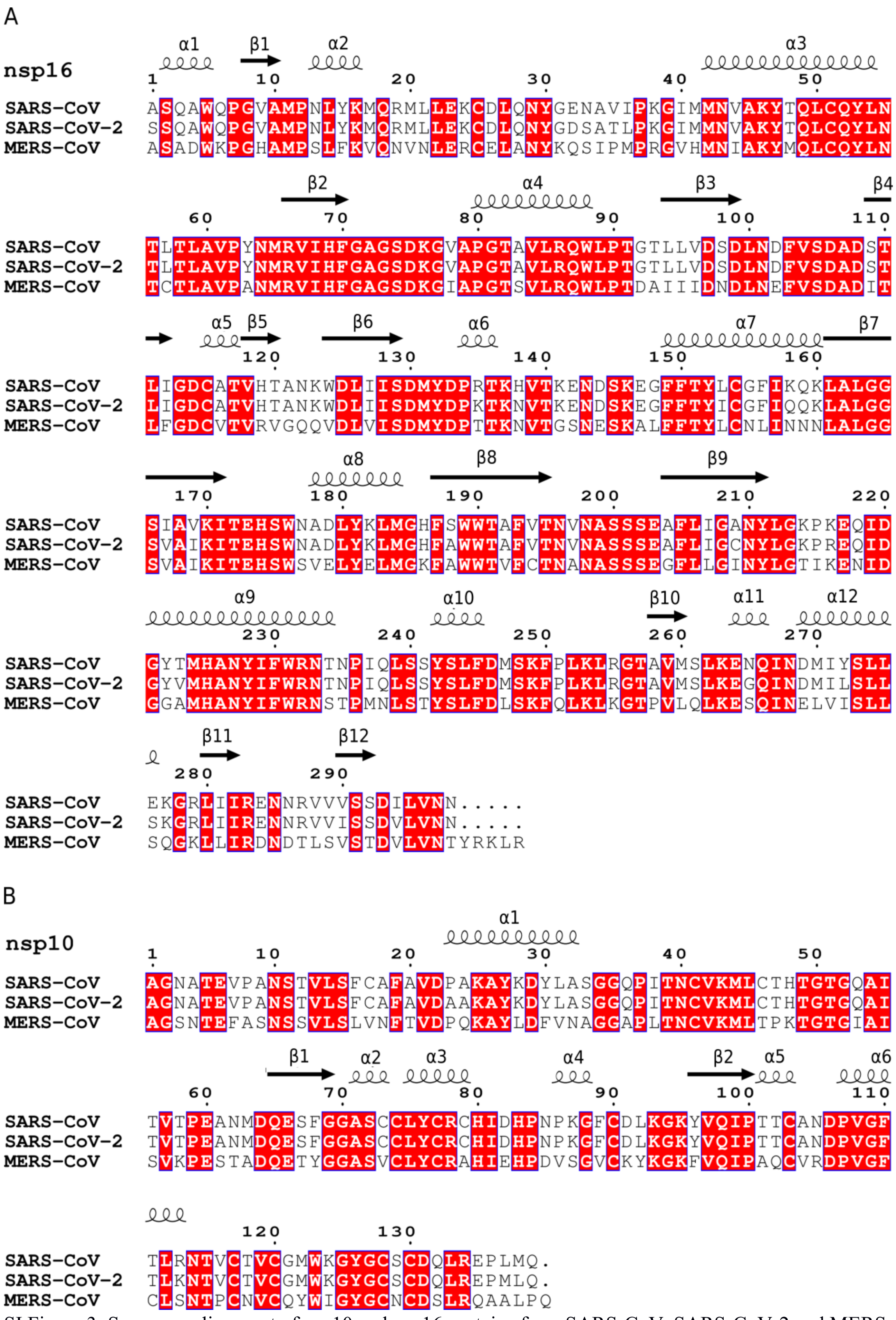

SI Figure 3: Sequence alignment of nsp10 and nsp16 proteins from SARS-CoV, SARS-CoV-2 and MERS-CoV.

Secondary structure elements of SARS-CoV-2 are displayed above the alignment. Conserved residues are highlighted in red boxes. Residues involved in formation of zinc fingers are depicted. Sequence alignment was rendered and analyzed by ESPript 3.0. For the alignment we used nsp10 and nsp16 sequences from SARS-CoV (NC_004718.3), SARS-2-CoV (NC_045512.2) and Mers-CoV (NC_019843.3).

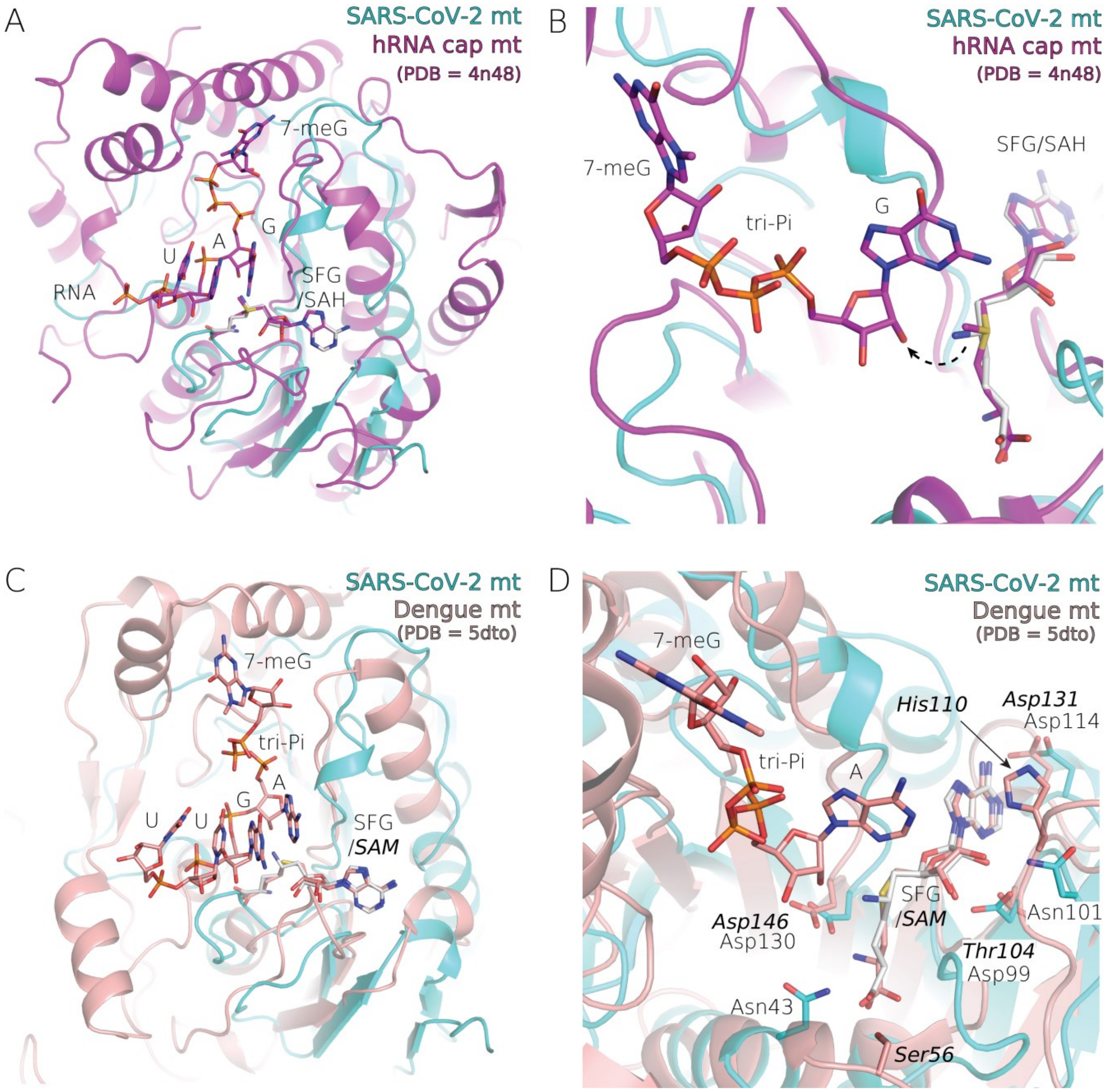

SI Figure 4: Structural alignments of SARS-CoV-2 with a human methyltransferase (4n48 - in magenta) and with a dengue virus methyltransferase (5dto - in lightpink)

A) Ligand (SFG/SAM) and m7GpppGAU B) Detailed view of the interaction with m7GpppG and SFG/SAM, similarly C) Ligand (SFG/SAH) and m7GpppAGUU with D) Detailed view of recognition of SFG/SAH (dengue amino acid residues are labeled oblique).
